# Update on the Epidemiology of Middle East Respiratory Syndrome Coronavirus — Worldwide, 2017–2023

**DOI:** 10.15585/mmwr.mm7419a1

**Published:** 2025-05-29

**Authors:** Anastasia S. Lambrou, Erin South, Claire M. Midgley, Chelsea Harrington, Lijuan Wang, Caelin Cubeñas, David Lowe, Glen R. Abedi, Cassandra Jones, Laura J. Hughes, Amber Winn, Melanie Wilkinson, Volha Katebi, Beth Schweitzer, Maria Van Kerkhove, Sophie von Dobschuetz, Leslie Edwards, Aron J. Hall, Cria O. Gregory, Hannah L. Kirking

**Affiliations:** ^1^Coronavirus and Other Respiratory Viruses Division, National Center for Immunization and Respiratory Diseases, CDC; ^2^General Dynamics Information Technology, Inc., Falls Church, Virginia; ^3^Division of Global Migration Health, National Center for Emerging and Zoonotic Infectious Diseases, CDC; ^4^Division of Laboratory Systems, Office of Laboratory Systems and Response, CDC; ^5^World Health Organization, Geneva, Switzerland.

**Summary**

**What is already known about this topic?**

Middle East respiratory syndrome (MERS) coronavirus (MERS-CoV) is a zoonotic virus transmitted sporadically from camels to humans, with limited subsequent human-to-human transmission. Most reported human cases of MERS have occurred in or near the Arabian Peninsula. Standardized clinical and epidemiologic criteria are used to determine who in the United States should be tested for MERS-CoV. In the United States, the last identified and confirmed MERS cases occurred in 2014.

**What is added by this report?**

Global reported MERS cases have declined substantially since the COVID-19 pandemic. Numbers of travelers entering the United States from in or near the Arabian Peninsula declined during the COVID-19 pandemic, but now have returned to prepandemic levels. U.S. MERS-CoV testing declined during 2017–2023 and remains low relative to prepandemic years. Clinical and epidemiologic criteria to guide U.S. testing were updated in 2024.

**What are the implications for public health practice?**

Though global reported MERS cases have declined substantially, continued MERS-CoV surveillance is important to maintaining MERS preparedness and response capabilities.

## Abstract

Middle East respiratory syndrome coronavirus (MERS-CoV) is a zoonotic virus transmitted sporadically from camels to humans. Most reported human Middle East respiratory syndrome (MERS) cases have occurred in or near the Arabian Peninsula. Limited human-to-human transmission can occur after close contact and has resulted in health care–associated outbreaks. Global reported MERS cases, U.S. testing data, and data on incoming U.S. travelers originating in and near the Arabian Peninsula during 2017–2023 were analyzed to guide U.S. MERS preparedness. Global MERS cases reported to the World Health Organization declined during the COVID-19 pandemic and remain substantially lower than during years preceding the pandemic. U.S. MERS-CoV testing numbers also declined and remain low relative to the prepandemic period. Although the number of travelers coming to the United States from in or near the Arabian Peninsula declined during the pandemic, incoming traveler volume returned to prepandemic levels. Further investigations are needed to determine whether the decline in global MERS cases reflects a true decrease in the number of infections, underascertainment of cases, or a combination. U.S. MERS persons under investigation criteria, standard clinical and epidemiologic characteristics used to guide who in the U.S. is tested for MERS-CoV, were updated in 2024 and can be used to guide clinicians and jurisdictional public health partners when considering MERS-CoV testing. Continued and targeted MERS-CoV material surveillance is important to maintaining preparedness and promptly responding to potential MERS cases.

## Introduction

CDC, World Health Organization (WHO), and global partners monitor Middle East respiratory syndrome (MERS) coronavirus (MERS-CoV) and its public health risk. MERS-CoV circulates among camel populations, and contact with camels has been associated with camel-to-human transmission (*1*). The virus was first detected in humans in 2012 (*2*). Human MERS-CoV infection can cause severe respiratory illness with an estimated case fatality rate of approximately 35%, and has been associated with limited household transmission and outbreaks in health care facilities (*3*).

Most human MERS-CoV infections result from camel-human interactions, with limited subsequent human-to-human transmission. Historically, the majority of camel and human cases have occurred in the Arabian Peninsula.* Outside this region, there is evidence of potential camel-to-human transmission in regions of Africa and of camel infection in South Asia^†^ (*4*–*6*). Travel-associated cases have occurred in at least 17 countries outside the Arabian Peninsula (*5*), leading to sporadic human-to-human transmission, such as in the large hospital-based outbreaks in 2015 in South Korea.^§^ In the United States, two confirmed, unrelated MERS^¶^ cases occurred in 2014, with no identified onward transmission; both cases occurred in health care workers who had recently traveled from Saudi Arabia (*7*). No fully approved vaccine currently exists for MERS-CoV, but a few candidate vaccines are in preclinical and early clinical trials. There is no specific MERS-CoV antiviral treatment, but active research and development are underway. Management currently includes supportive care and potential experimental treatment regimens.

Although there have been no reported U.S. MERS cases since 2014, current data do not support a reduction in the virologic prevalence of MERS-CoV in camels, and human MERS cases without camel exposure continue to occur (*4*).** Thus, CDC recommends MERS-CoV testing for persons within the United States who meet MERS person under investigation (PUI) criteria, which comprise specific combinations of clinical features and epidemiologic risks.^††^ When PUI criteria are met, indicating the need for testing, MERS-CoV testing is performed by Laboratory Response Network (LRN)^§§^ member laboratories or CDC.^¶¶^

The objectives of this report are to improve MERS awareness and preparedness by 1) updating previous CDC reports on global reported MERS cases (*7*,*8*) and 2) documenting U.S. specimens tested for MERS, estimated numbers of U.S. MERS PUIs, and estimated numbers of international travelers arriving to the U.S. from in or near the Arabian peninsula, during 2017–2023, stratified by pre–COVID-19 (January 2017–December 2019)*** and post–COVID-19 (January 2020–December 2023) surveillance periods because of the known effects of the pandemic on MERS testing and surveillance in the U.S. and worldwide.

## Methods

### Data Sources

Global MERS case report data, U.S. testing data, and data from U.S. incoming travelers originating in and near the Arabian Peninsula were analyzed and described by surveillance period. Reported global MERS case data (January 2017–November 2023) were obtained from WHO,^†††^ and case counts were described by reporting country and month. Case reporting dates were assigned as the date the first MERS-CoV–positive specimen was collected; if that date was missing, the WHO report date was used. 

U.S. MERS-CoV testing data were compiled from LRN and CDC to describe the total numbers of tests completed and total number of PUIs tested during January 2017–December 2023. Unique PUI identifiers were used; if these were unavailable, the number of PUIs was estimated, assuming that specimen results reported from a single laboratory with the same testing date corresponded to one person. Based on experience supporting state and local health departments managing MERS PUIs and current global MERS epidemiologic data, CDC updated the MERS PUI criteria used to guide testing in the U.S.

The numbers of incoming U.S. travelers arriving from in or near the Arabian Peninsula were described and used as a proxy for potential importation risk. Traveler data from Official Airline Guide (OAG) Traffic Analyzer^§§§^ were used; these data include modeled monthly aggregated numbers of total passengers originating from within or near the Arabian Peninsula and arriving in the United States during 2017–2023. Traveler data were stratified by country of travel origin and final U.S. arrival airport.

### Data Analysis

Data were combined and visualized using the statistical software R (version 4.1.3; R Foundation) to analyze temporal trends. Data were also graphed by origin and final arrival airport to geographically describe traveler volumes. This activity was reviewed by CDC, deemed research not involving human subjects, and was conducted consistent with applicable federal law and CDC policy.^¶¶¶^

## Results

### Global Reported MERS Cases

**FIGURE 1:**
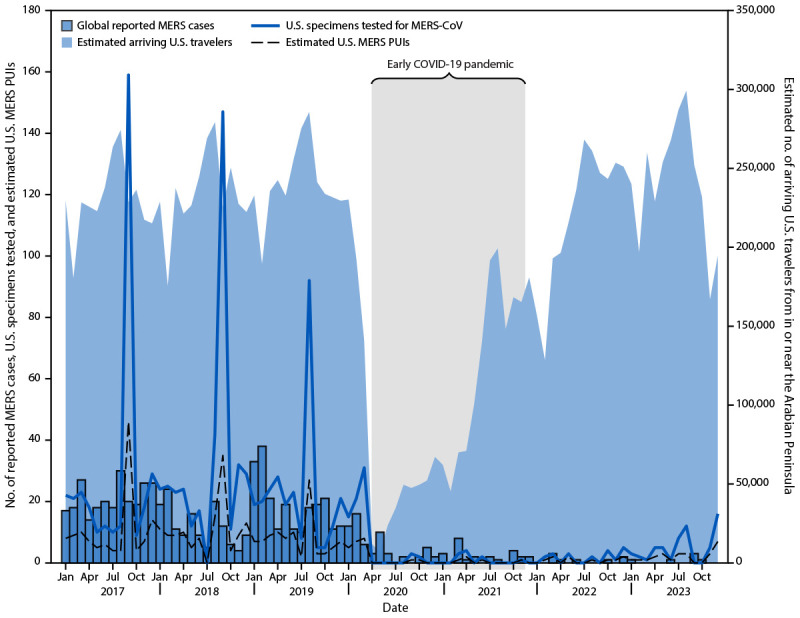
Global reported Middle East respiratory syndrome cases, number of U.S. patient specimens tested for Middle East respiratory syndrome coronavirus,* estimated number of U.S. Middle East respiratory syndrome persons under investigation, and estimated number of international travelers arriving in the United States from in or near the Arabian Peninsula^†^ — worldwide, January 1, 2017–December 31, 2023 **Abbreviations:** MERS = Middle East respiratory syndrome; MERS-CoV = MERS coronavirus; PUI = person under investigation. * Multiple specimens are often tested for each PUI; consequently, the estimated number of PUIs are also included in addition to the number of U.S. patient specimens tested for MERS-CoV. ^†^ Countries and regions considered in and near the Arabian Peninsula include Bahrain, Iran, Iraq, Israel, Jordan, Kuwait, Lebanon, Oman, Qatar, Saudi Arabia, Syria, United Arab Emirates, the West Bank and Gaza, and Yemen. No flights from the West Bank and Gaza to the United States were listed in the Official Airline Guide Traffic Analyzer during 2017–2023.

Since MERS case reporting commenced in 2012, a total of 2,608 cases have been reported to WHO as of December 31, 2023. Most cases have been detected in the Arabian Peninsula, with 2,200 (84%) occurring in Saudi Arabia. During 2017–2019, MERS case reporting was relatively stable, with a median of 224 cases reported each year (Table) (Figure 1). In 2020, the number of global reported MERS cases declined: a median of 17 cases per year have been reported during 2020–2023. Six cases were reported for 2023.

**TABLE:** Global reported Middle East respiratory syndrome cases, number of U.S. patient specimens tested* for Middle East respiratory syndrome coronavirus, estimated number of U.S. Middle East respiratory syndrome persons under investigation, and estimated number of international travelers arriving in the United States from in or near the Arabian Peninsula,^†^ across individual surveillance period years — worldwide, January 1, 2017–December 31, 2023

Year	Global reported no. of MERS cases	No. of U.S. specimens tested for MERS-CoV	Estimated no. of U.S. MERS PUIs	Estimated no. of international travelers arriving in the United States from in or near the Arabian Peninsula
**2017**	**253**	**343**	**124**	**2,759,995**
**2018**	144	386	128	2,808,009
**2019**	224	276	98	2,885,436
**Prepandemic period, 2017–2019 median (IQR)**	224 (184–239)	343 (310–365)	124 (111–126)	2,808,009 (2,784,002–2,846,723)
**2020**	61	72	22	906,783
**2021**	25	10	5	1,543,335
**2022**	8	20	10	2,650,384
**2023**	6	58	25	2,878,642
**Pandemic and postpandemic period, 2020–2023 median (IQR)**	17 (8–34)	39 (18–62)	16 (9–23)	2,096,860 (1,384,197–2,707,449)
**Total 2017–2023**	**721**	**1,165**	**412**	**16,432,584**
**Total median (IQR)^§^**	**61 ** **(17–184)**	**72** **(39–310)**	**25 ** **(16–111)**	**2,759,995 ** **(2,096,860–2,843,326)**

**Abbreviations: **MERS = Middle East respiratory syndrome; MERS-CoV = MERS coronavirus; PUI = person under investigation.

* Multiple specimens are often tested for each PUI; consequently, the estimated number of PUIs are also included in addition to the number of U.S. patient specimens tested for MERS-CoV.

^†^ Countries and regions considered in and near the Arabian Peninsula include Bahrain, Iran, Iraq, Israel, Jordan, Kuwait, Lebanon, Oman, Qatar, Saudi Arabia, Syria, United Arab Emirates, the West Bank and Gaza, and Yemen. No flights from the West Bank and Gaza to the United States were listed in the Official Airline Guide Traffic Analyzer during 2017–2023.

^§^ Median (IQR) during the years of the entire surveillance period (i.e., 2017–2023).

### U.S. MERS-CoV Testing

During the pre–COVID-19 pandemic period (2017–2019), a median of 343 specimens from an estimated median of 124 PUIs were tested annually for MERS-CoV in the United States (Table), with peak testing each year occurring during August and September. Annual Hajj pilgrimages to Mecca, Saudi Arabia, corresponded to this peak testing during 2017–2019. A substantial decline in U.S. specimens submitted for MERS-CoV testing began in April 2020, soon after declaration of the COVID-19 Public Health Emergency of International Concern,**** with no testing reported in the United States during April–August 2020. Limited specimen testing occurred during 2020–2023, with a median of 39 specimens from a median of 16 PUIs tested annually. In 2023, a total of 58 MERS-CoV specimens were tested in the United States (285 fewer than the prepandemic median of 343). No specimen has tested positive during the 2017–2023 surveillance period.

### Arabian Peninsula Travel to the United States

**FIGURE 2:**
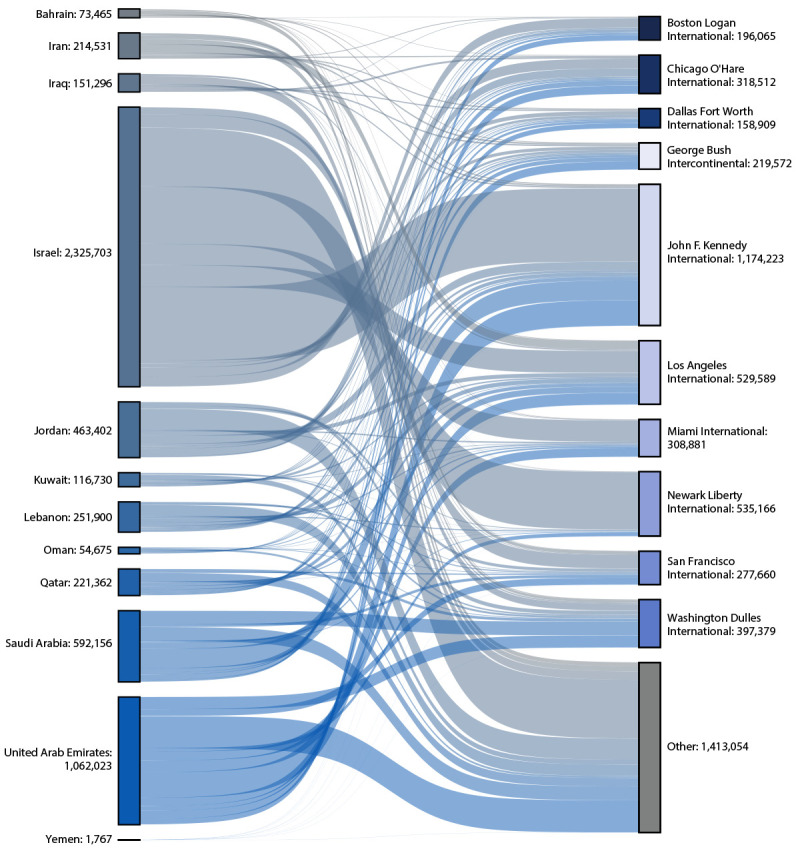
Estimated total number of travelers to the United States originating in and near the Arabian Peninsula,* by traveler volume — top 10 final U.S. arrival airports, January 1, 2022–December 31, 2023 * Countries and regions considered in and near the Arabian Peninsula include Bahrain, Iran, Iraq, Israel, Jordan, Kuwait, Lebanon, Oman, Qatar, Saudi Arabia, Syria, United Arab Emirates, the West Bank and Gaza, and Yemen. Estimated traveler volume from Syria were low (16) and therefore not included in this figure. No flights from the West Bank and Gaza to the United States were listed in the Official Airline Guide Traffic Analyzer during 2017–2023.

The estimated number of travelers to the United States who began their journey in or near the Arabian Peninsula remained consistent during 2017–2019 (median = 2,808,009 per year). During this time, peak travel each year occurred in July and August. Concurrent with the start of the COVID-19 pandemic, travel from these countries declined to an estimated 906,783 passengers in 2020^††††^ and then increased to 1,543,335 in 2021. The estimated number of international travelers to the United States from in or near the Arabian Peninsula increased to 2,650,384 in 2022 and to 2,878,642 through 2023, the highest annual estimate since 2019. Approximately one half of the estimated travelers to the United States from in or near the Arabian Peninsula during 2022–2023 arrived at 10 final U.S. arrival airports; 30.9% of all estimated travelers coming from the Arabian Peninsula arrived in New York City area airports (Figure 2).^§§§§ ^

## Discussion

This analysis of 2017–2023 MERS data facilitated comparisons of numbers of global cases, U.S. specimens tested, arriving U.S. travelers from countries in or near the Arabian Peninsula, and U.S. PUIs before and after the emergence of SARS-CoV-2. After the COVID-19 pandemic began, the average number of reported MERS cases declined sharply, with most cases still reported by Saudi Arabia.

It is possible that global reporting of MERS cases declined because cases were missed during the COVID-19 pandemic. The strain placed on health care and public health systems by the pandemic might also have limited identification of patients with risk factors for MERS; in addition, access to and volume of testing might have declined. These factors could have resulted in surveillance bias and declines in reported cases.

Other factors might be contributing to the decrease in reported global MERS cases, including pandemic mitigation measures, changes in interactions with camels,^¶¶¶¶^ potential cross-protection of antibodies against SARS-CoV-2 against MERS-CoV, and virologic changes (*9*,*10*); sustained submission of MERS-CoV genomic sequences to open-access databases is critical to identifying potential virologic changes (*10*). Further research and data-sharing are needed to better understand the causes of this large decrease in reported cases.

During 2017–2019, U.S. MERS-CoV testing volume and the total numbers of incoming travelers from in or near the Arabian Peninsula remained stable and exhibited summer peaks related to Hajj***** and travel. Both the number of incoming travelers to the United States and identification and testing of MERS PUIs decreased during the COVID-19 pandemic. However, although international travel has largely returned to prepandemic levels, the number of U.S. specimens submitted for MERS-CoV testing has remained low. If the proportion of persons who meet PUI clinical criteria were to remain constant, U.S. MERS-CoV testing would be expected to be higher to mirror the increases in travelers. Furthermore, understanding where travelers arrive in the United States might help guide state and local health departments concerning the possible risks and need for MERS-CoV testing. 

The findings in this report might help to guide MERS preparedness priorities and activities. The decrease in the number of global MERS cases, and the potential causes for this decline, should be further investigated, including through surveillance evaluations, immunologic studies, and genomic sequencing. Despite decreases in globally reported human MERS cases, the United States remains at possible risk for MERS. Data do not support reduction in the virologic prevalence of MERS-CoV in camels. Ensuring that a comprehensive One Health approach, connecting human, animal, and environmental health, is taken to assess the risk for MERS globally and within the United States is vital to maintaining adequate preparedness activities. In light of these considerations, traveler and testing data can provide information regarding testing needs, testing capacity, and appropriate surveillance strategies. More specifically, jurisdictions with airports receiving high volumes of travelers from in or near the Arabian Peninsula are strategic locations for strengthening MERS testing and surveillance approaches. 

In 2024, CDC released updated criteria for testing MERS PUIs for MERS-CoV infection (Diagnostic Testing for MERS | MERS | CDC) to incorporate 1) emergence of SARS-CoV-2 as another cause of severe respiratory illness, 2) increased use of multiplex pathogen detection platforms, and 3) evidence related to potential MERS-CoV spillover from camels to humans in parts of Africa (*4*,*5*). These updates included clarification of clinical criteria and the expansion of epidemiologic risks criteria to include 1) direct camel contact in or near the Arabian Peninsula as a risk factor for those with mild illness, 2) direct camel contact among patients with severe presentation and recent travel to regions of Africa,^†††††^ and 3) occupational exposure among laboratorians or researchers handling infectious MERS-CoV material.^§§§§§^ U.S. clinicians should obtain a thorough travel history from patients with acute respiratory illness of unknown etiology, and work with their jurisdictional public health departments to obtain MERS testing for patients who meet MERS PUI testing criteria. CDC will continue to maintain and update MERS PUI testing criteria as new information emerges. Further MERS materials and guidance is available on CDC and WHO websites.^¶¶¶¶¶^

### Limitations

The findings in this report are subject to at least four limitations. First, MERS cases reported to WHO reflect data submitted by member nations through the 2005 International Health Regulations mechanism; data completeness and quality vary. Second, the U.S. MERS-CoV testing data include minimal metadata, thus limiting epidemiologic and PUI analyses. Third, OAG data are modeled using ticket sales, which might not reflect the true number of travelers. Finally, traveler origin country is a proxy for countries where MERS-CoV is likely endemic; it does not identify other risk factors.

### Implications for Public Health Practice

Epidemiologic, testing, and traveler data are indicators that are essential to guiding public health investigations and readiness activities. Strengthening MERS-CoV surveillance and ongoing risk assessments are critical to supporting MERS-CoV and more broadly novel coronavirus pandemic preparedness and surveillance. Continued and targeted MERS-CoV surveillance is important to maintaining preparedness and promptly responding to potential MERS cases.

## Footnotes

* Countries and regions considered in and near the Arabian Peninsula include Bahrain, Iran, Iraq, Israel, the West Bank and Gaza, Jordan, Kuwait, Lebanon, Oman, Qatar, Saudi Arabia, Syria, United Arab Emirates, and Yemen.

† Camel-to-human transmission is most commonly reported in countries in and near the Arabian Peninsula, but evidence also exists for possible infection in Africa and South Asia. SeroTracker (Accessed 5 May 2023).

§ The largest MERS-CoV outbreak outside countries in and near the Arabian Peninsula occurred in South Korea in 2015 with 186 confirmed cases seeded from a single travel-associated case resulting in a large nosocomial-driven outbreak. About Middle East Respiratory Syndrome (MERS) | MERS | CDC

¶ MERS is a viral respiratory illness caused by MERS-CoV.

**
Middle East respiratory syndrome coronavirus - Kingdom of Saudi Arabia

†† CDC MERS PUI testing criteria and diagnostic testing information is available online. Diagnostic Testing for MERS | MERS | CDC

§§ MERS-CoV testing and PUI clinical data are not required to be reported to CDC, but MERS-CoV testing results are required to be reported through LRN. About The Laboratory Response Network | The Laboratory Response Network Partners in Preparedness | CDC

¶¶ All MERS diagnostic testing in the United States is completed at public health laboratories, per the diagnostic assays’ Food and Drug Administration’s Emergency Use Authorization.

*** 2017 was selected as the earliest surveillance year because of 1) the need to update MERS epidemiology since previous *MMWR* reports, 2) need for a pre–COVID-19 pandemic analysis and comparison period, and 3) January 2017 being the earliest month and year that traveler data were available.

††† Reporting of MERS cases to WHO is required under International Health Regulations. International Health Regulations

§§§ OAG estimates use ticket sales and reporting from airline carriers to model 100% of the airline travel market. OAG estimated adjusted travel volume represents the aggregate number of passenger journeys, not necessarily unique individual travelers (version 2.8.0; OAG Aviation Worldwide Ltd. OAG Traffic Analyzer). These data are commercial, proprietary data, are under license, and are not publicly available.

¶¶¶ 5 C.F.R. part 46.102(l)(2), 21 C.F.R. part 56; 42 U.S.C. Sect.241(d); 5 U.S.C.0 Sect.552a; 44 U.S.C. Sect. 3501 et seq.

**** Public Health Emergency of International Concern. WHO Coronavirus disease (COVID-19) pandemic

†††† Because of the COVID-19 pandemic, in 2020, Hajj was held with crowd control and travel restrictions (July 28–August 2). Hajj resumed usual pilgrimage activities in 2021 with the following dates: July 17–21, 2021; July 7–12, 2022; and June 26–July 1, 2023. Hajj results in increased MERS-CoV testing (because of increased travel and nonspecific respiratory symptoms), but as of the release of this report there have been no cases of MERS reported in association with Hajj.

§§§§ In descending order of total number of passengers: John F. Kennedy International Airport (New York City area; 1,174,223), excluding travelers from Syria due to low numbers; Newark Liberty International Airport (New York City area; 535,166); Los Angeles International Airport, (529,589); Washington Dulles International Airport, (397,379); Chicago O’Hare International Airport, (318,512), excluding travelers from Syria due to low numbers; Miami International Airport, (308,881); San Francisco International Airport, (277,660); George Bush Intercontinental Airport, (219,572); Boston Logan International Airport, (196,065); and Dallas Fort Worth International Airport, (158,909).

¶¶¶¶ For example, the nature and frequency of human interaction with camels such as feeding, slaughtering, riding, and coming in direct contact might have decreased during the COVID-19 pandemic because of travel restrictions, restricted movements, mobility changes, and other global interventions.

***** Hajj dates of *MMWR* surveillance data period: August 30–September 4, 2017; August 19–24, 2018; and August 9–14, 2019.

††††† Because the risk for MERS-CoV transmission from camels in North, West, and East Africa is not yet fully understood, MERS evaluation should be considered for travelers coming from these regions who develop severe respiratory illness within 14 days of direct camel contact.

§§§§§ Diagnostic and research facilities that handle MERS-CoV should have established procedures instructing their staff members in how to prevent and respond to occupational exposures. Laboratory exposure might occur through contact with infected animals and viral specimens without proper precautions and personal protective equipment.

¶¶¶¶¶ WHO MERS Outbreak Toolbox; WHO Middle East respiratory syndrome coronavirus (MERS-CoV)

## References

[1] Killerby ME, Biggs HM, Midgley CM, Gerber SI, Watson JT. Middle East respiratory syndrome coronavirus transmission. Emerg Infect Dis 2020;26:191–8. 10.3201/eid2602.190697 31961300 PMC6986839

[2] Zaki AM, van Boheemen S, Bestebroer TM, Osterhaus AD, Fouchier RA. Isolation of a novel coronavirus from a man with pneumonia in Saudi Arabia. N Engl J Med 2012;367:1814–20. 10.1056/nejmoa1211721 23075143

[3] Alzahrani A, Kujawski SA, Abedi GR, Surveillance and testing for Middle East respiratory syndrome coronavirus, Saudi Arabia, March 2016–March 2019. Emerg Infect Dis 2020;26:1571–4. 10.3201/eid2607.200437 32568049 PMC7323557

[4] Ogoti BM, Riitho V, Wildemann J, Biphasic MERS-CoV incidence in nomadic dromedaries with putative transmission to humans, Kenya, 2022–2023. Emerg Infect Dis 2024;30:581–5. 10.3201/eid3003.231488 38407189 PMC10902546

[5] Arabi YM, Balkhy HH, Hayden FG, . Middle East respiratory syndrome. N Engl J Med. 2017;376(6):584–94. 10.1056/nejmsr1408795 28177862 PMC5362064

[6] Islam A, Epstein JH, Rostal MK, Middle East respiratory syndrome coronavirus antibodies in dromedary camels, Bangladesh, 2015. Emerg Infect Dis 2018;24:926–8. 10.3201/eid2405.171192 29664373 PMC5938793

[7] Bialek SR, Allen D, Alvarado-Ramy F, ; CDC. First confirmed cases of Middle East respiratory syndrome coronavirus (MERS-CoV) infection in the United States, updated information on the epidemiology of MERS-CoV infection, and guidance for the public, clinicians, and public health authorities—May 2014. MMWR Morb Mortal Wkly Rep 2014;63:431–6. 24827411 PMC5779407

[8] Rha B, Rudd J, Feikin D, ; CDC. Update on the epidemiology of Middle East respiratory syndrome coronavirus (MERS-CoV) infection, and guidance for the public, clinicians, and public health authorities—January 2015. MMWR Morb Mortal Wkly Rep 2015;64:61–2. 25632953 PMC4584559

[9] Zedan HT, Smatti MK, Thomas S, Assessment of broadly reactive responses in patients with MERS-CoV infection and SARS-CoV-2 vaccination. JAMA Netw Open 2023;6:e2319222. 10.1001/jamanetworkopen.2023.19222 37389876 PMC10314312

[10] Hassan AM, Mühlemann B, Al-Subhi TL, Ongoing evolution of Middle East respiratory syndrome coronavirus, Saudi Arabia, 2023–2024. Emerg Infect Dis 2025;31:57–65. 10.3201/eid3101.241030 39641462 PMC11682817

